# Interleukin‐1β augments the angiogenesis of endothelial progenitor cells in an NF‐κB/CXCR7‐dependent manner

**DOI:** 10.1111/jcmm.15220

**Published:** 2020-04-02

**Authors:** Xia Fan, Luqing He, Qiaoxia Dai, Junhong He, Xiangjuan Chen, Xiaozhen Dai, Chi Zhang, Da Sun, Xue Meng, Shiyue Sun, Jiameng Huang, Jun Chen, Lin Lin, Liangmiao Chen, Yi Tan, Xiaoqing Yan

**Affiliations:** ^1^ Department of Pharmacy Chinese‐American Research Institute for Diabetic Complications Wenzhou Medical University Wenzhou China; ^2^ Department of Endocrinology The Second Affiliated Hospital of Wenzhou Medical University Wenzhou China; ^3^ Department of Obstetrics and Gynecology The first affiliated Hospital of Wenzhou Medical University Wenzhou China; ^4^ School of Biomedicine Chengdu Medical College Chengdu China; ^5^ Department of Endocrinology The Third Affiliated Hospital of Wenzhou Medical University Wenzhou China; ^6^ Institute of Life Sciences Wenzhou University Wenzhou China; ^7^ School of Nursing Wenzhou Medical University Wenzhou China; ^8^ Department of Obstetrics and Gynecology The Third Affiliated Hospital of Wenzhou Medical University Wenzhou China

**Keywords:** angiogenesis, CXC chemokine receptor 7, endothelial progenitor cells, extracellular signal‐regulated kinase‐1/2, interleukin‐1β

## Abstract

Endothelial progenitor cells (EPCs) are able to trigger angiogenesis, and pro‐inflammatory cytokines have beneficial effects on angiogenesis under physiological and pathological conditions. C‐X‐C chemokine receptor type 7 (CXCR‐7), receptor for stromal cell‐derived factor‐1, plays a critical role in enhancing EPC angiogenic function. Here, we examined whether CXCR7 mediates the pro‐angiogenic effects of the inflammatory cytokine interleukin‐1β (IL‐1β) in EPCs. EPCs were isolated by density gradient centrifugation and angiogenic capability was evaluated in vitro by Matrigel capillary formation assay and fibrin gel bead assay. IL‐1β elevated CXCR7 expression at both the transcriptional and translational levels in a dose‐ and time‐dependent manner, and blockade of the nuclear translocation of NF‐κB dramatically attenuated the IL‐1β‐mediated up‐regulation of CXCR7 expression. IL‐1β stimulation significantly promoted EPCs tube formation and this effect was largely impaired by CXCR7‐siRNA transfection. IL‐1β treatment stimulated extracellular signal‐regulated kinase 1/2 (Erk1/2) phosphorylation, and inhibition of Erk1/2 phosphorylation partially impaired IL‐1β‐induced tube formation of EPCs but without significant effects on CXCR7 expression. Moreover, blocking NF‐κB had no significant effects on IL‐1β‐stimulated Erk1/2 phosphorylation. These findings indicate that CXCR7 plays an important role in the IL‐1β‐enhanced angiogenic capability of EPCs and antagonizing CXCR7 is a potential strategy for inhibiting angiogenesis under inflammatory conditions.

## 1 INTRODUCTION

Endothelial progenitor cells (EPCs)are a kind of endothelial precursor cells that have the potential to differentiate into a mature endothelial cell and contribute to endothelial generation and vessel repair in ischaemic tissues and injured blood vessel endothelium, respectively.^1^ EPCs control the angiogenic switch of many physiological and pathologic processes, such as the neovascularization of ischaemic tissues^2^, ^3^, ^4^ and tumorigenesis.^5^ Local or systemic transplantation of EPCs derived from bone marrow,^6^ peripheral blood^7^ or cord blood^8^ can enhance ischaemic angiogenesis and improve the function of ischaemic tissues in animals with limb or myocardial ischaemia.

C‐X‐C chemokine receptor type 7 (CXCR‐7) is a seven‐transmembrane G‐protein‐coupled receptor that is widely expressed in the haematopoietic system,^9^ cardiac microvessels, brain,^10^ kidney^11^ and several tumour cell lines.^12^, ^13^, ^14^ CXCR7 is a novel alternative receptor for stromal cell‐derived factor 1 (SDF‐1) and has an approximately 10 times higher binding affinity with SDF‐1 than CXCR4.^15^ CXCR7 knockout mice died at birth because of ventricular septal defects and semilunar heart valve malformation, and this phenotype can be recapitulated in mice with an endothelium‐specific deletion of CXCR7, which indicates that CXCR7 is essential for valve formation, vessel protection, endothelial cell growth and survival.^16^ In addition, CXCR7 has been reported to be a co‐receptor for human and simian immunodeficiency viruses^17^ and to be related to memory B cell differentiation.^18^ This receptor also uniquely mediates SDF‐1‐induced renal progenitor cell survival and adhesion to endothelial cells.^19^ CXCR7 can also shape the distribution of the chemokine SDF‐1 in the environment by acting as a decoy receptor in zebrafish posterior lateral line development.^20^, ^21^ Moreover, CXCR7 was reported to enhance tumour development^13^, ^22^, ^23^, ^24^, ^25^ and metastasis^22^, ^26^ and to be up‐regulated in tumour‐associated vessels,^12^ indicating a potential role in tumour angiogenesis. Our previous studies^27^, ^28^ also demonstrated that CXCR7 plays a critical role in the SDF‐1 promotion of EPC‐mediated angiogenesis. CXCR7 mediates EPC adhesion, trans‐endothelial migration and tube formation induced by SDF‐1 and CXCR4, and exclusively mediates EPC survival. In addition, CXCR7 is able to enhance vascular endothelial growth factor (VEGF) expression levels in an SDF‐1‐independent manner.^29^ All these facts show that CXCR7 plays a critical role in EPC‐mediated angiogenesis.

Interleukin‐1β (IL‐1β) is a potent immunoregulatory and pro‐inflammatory cytokine secreted by a variety of activated immune cells. IL‐1β can infiltrate solid tumours and has been shown to be a pro‐angiogenic factor in solid tumours.^30^ IL‐1β can up‐regulate VEGF expression in tumour cells and augment angiopoietin‐1 expression in human endothelial cells.^31^ Moreover, Rosell et al^32^ found that IL‐1β augments the angiogenic responses of murine EPCs in vitro in an Erk1/2‐dependent manner. However, inhibiting the Erk1/2 pathway can only partly suppress EPC tube formation.^32^ This fact indicates that there might be some other mechanism involved in the IL‐1β‐mediated angiogenic capability of EPCs. In the present study, we investigated the role of CXCR7 in the IL‐1β‐promoted angiogenic capability of EPCs.

## 2 MATERIALS AND METHODS

### 2.1 Reagents

Recombinant human IL‐1β was purchased from Peprotech. Histopaque 1077 (Sigma), EGM‐2 (Lonza), human fibronectin (BD Biosciences) and foetal bovine serum (FBS, Life Techonologies) were used for EPC isolation and culture. Mouse anti‐CD133 antibody (Miltenyi Biotec), rabbit polyclonal antibody against VEGFR‐2 (BD Biosciences), DiI‐acetylated low‐density lipoprotein (Biomedical Technologies) and Ulex europaeus agglutinin‐1(Sigma) were used for EPC identification. Matrigel was purchased from BD Biosciences for the Matrigel capillary formation assay. Chemical inhibitors including the phospho‐Erk1/2 inhibitor U0126 (MedChem Express) and BAY 11‐7082 (Beyotime) were included in the mechanistic study. In fibrin gel bead assay, cytodex‐3 beads were purchased from GE Healthcare, while aprotinin, type I fibrinogen and thrombin were purchased from Sigma.

CXCR7, phospho‐Erk1/2, total‐Erk1/2, NF‐κB, histone H3 and β‐actin protein expression levels were detected by Western blot assay using primary antibodies including anti‐CXCR7 (Proteintech, 1:1000), anti‐phospho‐Erk1/2(Cell Signaling Technology, 1:5000), anti‐totalErk1/2 (Cell Signaling Technology, 1:5000), anti‐NF‐κB (Cell Signaling Technology, 1:1000), anti‐histone H3 (Santa Cruz, 1:1000) and anti‐β‐actin (Santa Cruz, 1:10 000) antibodies and the secondary HRP‐conjugated antibodies including goat anti‐rabbit IgG (Santa Cruz) and donkey anti‐goat IgG (Santa Cruz). The PE‐conjugated CXCR7 monoclonal antibody (Biolegend) and its corresponding IgG isotype control (Biolegend) were used for flow cytometry.

RNA Extraction Kit (Bioteke), High‐Capacity cDNA Reverse Transcription Kit (Applied Biosystems), SYBR Green PCR Master Mix Kit (Applied Biosystems), CXCR7 and β‐actin primers synthesized by Applied Biosystems were used in quantitative real‐time PCR (qRT‐PCR).

### 2.2 Human cord blood EPC isolation and identification

Human umbilical cord blood samples (20‐40 mL) from four healthy newborns (three boys and one girl; gestational age range, 38‐40 weeks) were collected in CPD solution. The Institutional Review Board in The Third Affiliated Hospital of Wenzhou Medical University approved all protocols and informed consent was obtained from all the parents of the newborns. EPCs were isolated from human cord blood as previously described.^27^ In brief, cord blood (20‐40 mL) was diluted 1:1 with Hanks balanced salt solution (HBSS; Invitrogen), overlaid onto an equivalent volume of Histopaque 1077 and centrifuged at 400 *g* for 30 minutes at room temperature. Mononuclear cells (MNCs) were isolated, washed three times with EGM‐2 and then plated into one well of a six‐well plate coated with human fibronectin (2 mg/cm^2^) in EGM‐2 supplemented with 10% FBS. The plate was incubated at 37°C in a humidified environment with 5% CO_2_. After 24 hours, the unattached cells and debris were removed by washing with medium. The medium was changed daily for 7 days and thereafter on alternate days. At day 21, EPCs were characterized using acetylated low‐density lipoprotein uptake and a lectin binding assay. First, cells were incubated with DiI‐acetylated low‐density lipoprotein (Dil‐acLDL, final concentration 10 mg/mL) at 37°C for 4 hours and then fixed with 3% paraformaldehyde for 10 minutes. After two washes with phosphate‐buffered saline (PBS), the cells were then incubated with Ulex europaeus agglutinin‐1 (UEA‐1, final concentration 10 mg/mL) for 1 hour. After staining, pictures were taken with a fluorescence microscope (Olympus IX71, Olympus). Double‐positive‐stained cells were identified as differentiating EPCs. EPCs were further identified by CD133 and vascular endothelial growth factor receptor 2 (VEGFR‐2) expression using immunofluorescent staining. In this assay, mouse anti‐CD133 antibody and rabbit polyclonal antibody against VEGFR‐2 were used.

### 2.3 SiRNA transfection

Human CXCR7 siRNAs (siRNA349, sense 5′‐CGCUCUCCUUCAUUUACAUTT‐3′, anti‐sense 5′‐AUGUAAAUGAAGGAGAGCGTT‐3′; siRNA877, sense 5′‐CCGUUCCCUUCU CCAUUAUTT‐3′, anti‐sense 5′‐AUAAUGGAGAAGGGAACGGTT‐3′; and siRNA1197, sense 5′‐GCCUUCAUCUUCAAGUACUTT‐3′, anti‐sense 5′‐AGUACUUGAAGAUGAAGGCTT‐3′), human Erk1‐siRNA (sense CGAGAUCUAAAGCCCUCCATT, anti‐sense UGGAGGGCUUUAGAUCUCGTT), human Erk2‐siRNA (sense GUCCAUUGAUAUUUGGUCUTT, anti‐sense AGACCAAAUAUCAAUGGACTT) and corresponding control siRNA (ctrl siRNA) were purchased from GenePharma (Shanghai, China). SiRNA transfection was performed using Lipofectamine 3000 (Invitrogen) according to the manufacturer's instructions. Briefly, EPCs were cultured in 24‐well plates to 70%‐90% confluence. Lipofectamine 3000 (1.5 μL) and 20 pmol siRNA (CXCR7 siRNA or Ctrl siRNA) were diluted with 25 mL of Opti‐MEM, respectively. Diluted Lipofectamine 3000 was added to the siRNA solution and incubated for 5 minutes at room temperature. Next, the siRNA‐Lipofectamine mixture was added to the EPCs and incubated for 2 days, and then, the CXCR7 mRNA expression level was evaluated by qRT‐PCR. Transfected EPCs were used for the tube formation assay within 1 week after transfection.

### 2.4 Detecting Erk1/2 phosphorylation, CXCR7, nuclear NF‐κB and histone H3 expression by Western blot

Endothelial progenitor cells were washed with pre‐cooled PBS and lysed in RIPA buffer solution (Cell Signaling Technology) containing phosphatase inhibitor and protease inhibitor (Roche). Cell lysates were clarified by centrifugation at 15 400 *g* at 4°C for 30 minutes, and protein concentrations were determined using the Quick Start Bradford Dye Reagent (Bio‐Rad). Proteins (30 μg per sample) were prepared by adding loading buffer and DTT and were denatured by incubating at 95°C for 5 minutes; then, the samples were loaded onto a 10% SDS‐PAGE gel. After electrophoresis, proteins were transferred onto a nitrocellulose membrane (Merck Millipore). The membranes were incubated overnight at 4°C with rabbit anti‐CXCR7 antibody or rabbit anti‐phosphorylated‐Erk1/2 after blocking in 5% skim milk for 1 hour. Then, the membranes were washed with Tris‐buffered saline with Tween‐20 (TBST) three times and incubated with the corresponding HRP‐conjugated secondary antibody at room temperature for 1 hour. After three washes with TBST, the bands were visualized using ECL and detected by a Western blot imaging system (Tanon). For total‐Erk1/2 detection, the same membrane that was used for phosphorylated Erk1/2 detection was washed with stripping buffer (Signagen Laboratories) for 10 minutes, blocked with 5% skim milk and incubated with an anti‐Erk1/2 antibody following the same procedure described above.

To detect the nuclear content of NF‐κB and histone H3, the nuclear protein from EPCs was extracted using a nucleoprotein extraction kit (Beyotime) according to the manufacturer's instructions. The nuclear protein concentration was measured by a BCA Protein Assay Kit (Beyotime).

### 2.5 Detecting CXCR7 mRNA expression by real‐time PCR

Total mRNA was extracted from each different group of EPCs using an RNA Extraction Kit, and the concentration was determined with a Nanodrop2000 (Thermo Scientific). Then, the mRNA was reverse transcribed to cDNA with a High‐Capacity cDNA Reverse Transcription Kit (Thermo Fisher Scientific). qRT‐PCR was performed using a SYBR Green PCR Master Mix Kit according to the manufacturer's instructions in a 7500 Real‐Time PCR machine (Applied Biosystems). The specific primers for CXCR7 and β‐actin were purchased from Applied Biosystems. The house‐keeping gene β‐actin was used as an internal loading control.

### 2.6 Detecting CXCR7 Expression by flow cytometry assay

Endothelial progenitor cells were cultured in six well plates to 50%‐70% confluence. Then, culture media were changed to EGM‐2 with or without IL‐1β at different doses ranging from 0.001, 0.01, 0.1, 0.5, 1, 5, 10, 50 to 100 ng/mL in the dose‐effect study or 10 ng/mL IL‐1βin the time‐course study. After treatment, EPCs were detached and resuspended in PBS containing 1% FBS. The cell surface expression of CXCR7 was detected using a PE‐conjugated CXCR7 monoclonal antibody (Biolegend) and corresponding IgG isotype control. Data were acquired on a FC500 flow cytometer (Beckman Coulter) using CXP cytometer software (Beckman Coulter) and analysed using CXP analysis software (Beckman Coulter). At least 10 000 cells were analysed per sample.

### 2.7 Matrigel capillary formation assay

The Matrigel capillary formation assay was performed as previously described.^3^, ^28^ Briefly, growth factor‐reduced Matrigel (BD Biosciences) was thawed in ice water overnight, and then, 130 μL of Matrigel was added to a pre‐cooled 48‐well plate and incubated at 37°C for 30 minutes to polymerize. EPCs were starved in serum‐free MCDB131 medium for 12 hours to remove the pro‐angiogenic growth factor activity from the EGM‐2 incubation. Next, EPCs (4 × 10^4^ cells per well) were seeded onto Matrigel in the presence or absence of 10 ng/mL IL‐1β or 10 μmol/LErk1/2 inhibitor U0126. After 16 hours, capillaries were recorded in a blinded manner under a Leica DMI3000B light microscope (Leica) equipped with a digital camera (Olympus DP25). The length of the capillaries in the images was measured using ImageJ (http://rsbweb.nih.gov/ij/). At least six fields of view were examined per well.

### 2.8 Fibrin gel bead assay

The tube formation capability of EPCs was evaluated by fibrin gel bead assay according to the protocol of Nakatsu et al^33^ Briefly, Cytodex‐3 beads were washed with 1 mL of warm EGM‐2. EPCs or EPCs transfected with Ctrl siRNA, CXCR7‐siRNA or Erk siRNA were used for coating beads. EPCs (1 × 10^6^ cells) were mixed with 2500 beads in 1.5 mL of warm EGM‐2 medium in a FACS tube and then incubated for 4 hours at 37°C with shaking every 20 minutes. Thereafter, EPC‐coated beads were transferred to a T25 flask in 5 mL of EGM‐2 and incubated overnight. After being washed three times with EMG‐2, EPC‐coated beads were resuspended in 2.0 mg/mL fibrinogen solution containing 0.15 units/mL aprotinin at a concentration of ~500 beads/mL. Thrombin (0.625 Units/mL) was added to each well of a 24‐well plate, and then, 0.5 mL of the fibrinogen/bead suspension was added to thrombin and mixed gently. The plate was left in the hood for 5 minutes to let the beads settle and then placed in a 37°C incubator for 10‐15 minutes to promote clotting. MCDB131 (1 mL) containing 5% FBS with or without 10 ng/mL IL‐1β was added to each well. Tube‐like structures were recorded in a blinded manner under a Leica DMI3000B light microscope (Leica) equipped with a digital camera (Olympus DP25). The length of the tube‐like structures in the images was measured using ImageJ (http://rsbweb.nih.gov/ij/). At least 10 beads were examined per well. To evaluate the alterations induced by the Erk inhibitor U0126 on the IL‐1β‐mediated the tube formation capability of EPCs, beads coated with normal EPCs were cultured in MCDB131 medium containing 5% FBS with or without 10 ng/mL IL‐1β and/or 10 μmol/L Erk1/2 inhibitor U0126.

### 2.9 Statistical analysis

Results were obtained from at least three independent experiments and are presented as the means ± SD. Statistical analyses were performed using Original 9.0 software (Original Lab) with Student's *t* test or one‐way ANOVA, followed by post hoc multiple comparisons with the Scheffe test. Statistical significance was set at *P* < .05.

## 3 RESULTS

### 3.1 Identification of human cord blood EPCs

**Figure 1:**
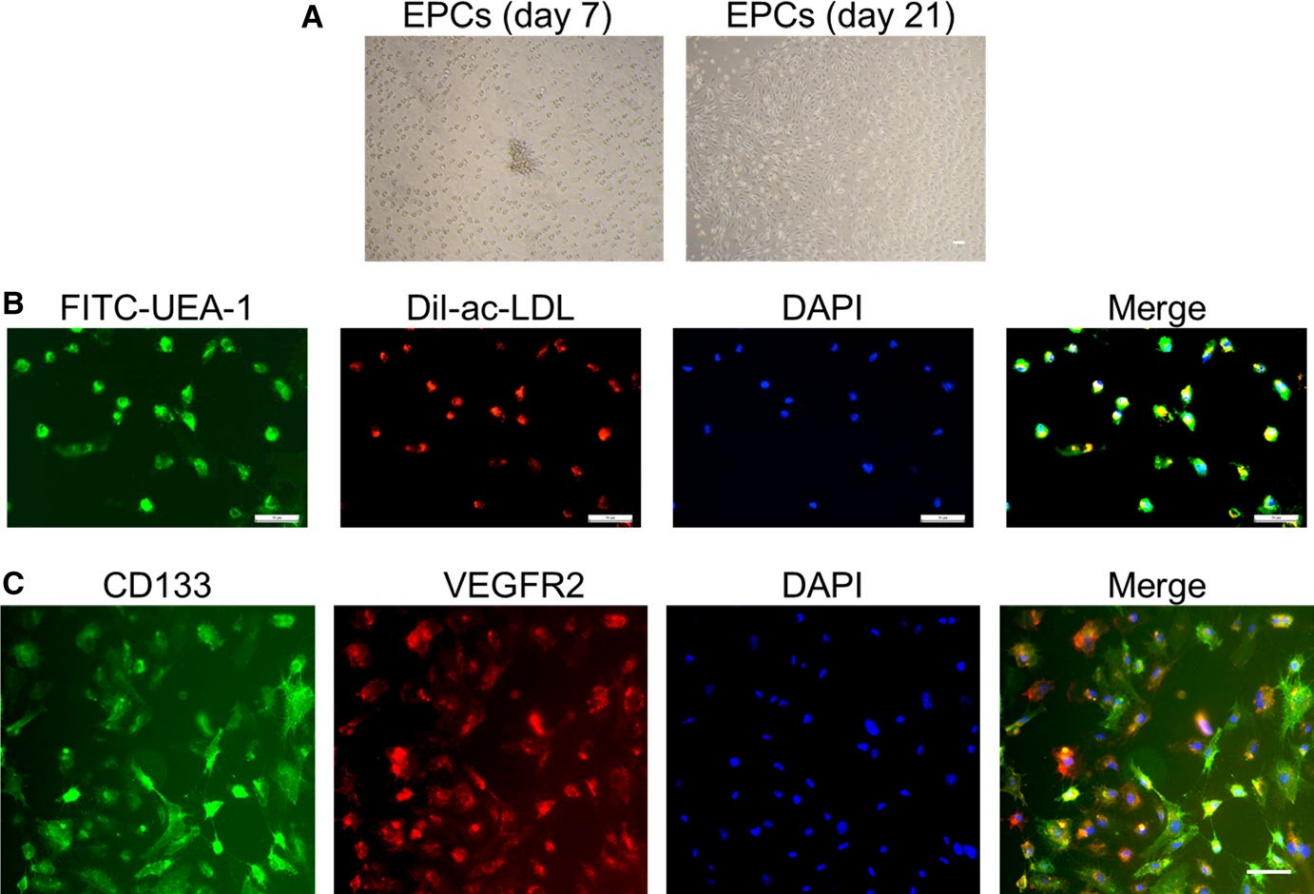
Identification of human cord blood EPCs. A, typical cell cluster appeared after 7 days of culture in EGM‐2 (left) and different morphologies emerged over 14‐21 days of culture (right). B, DiI‐acLDL uptake and FITC‐UEA‐1 binding assays showed that cells were both DiI‐acLDL and FITC‐UEA‐1 positive, indicating that the isolated MNCs are EPCs. C, Cultured MNCs were further characterized by immunofluorescent staining using the EPC‐specific markers CD133/VEGFR‐2, and the nucleus was indicated by DAPI staining. Scale bars represent 50 μm

Endothelial progenitor cells isolated from human cord blood were cultured with EGM‐2 in plates coated with fibronectin. Typical cell clusters appeared at day 7, and colonies emerged after 21 days of culture (Figure 1A). Acetylated low‐density lipoprotein uptake and the lectin binding assay showed that these cells can uptake DiI‐acLDL and bind with UEA‐1 (Figure 1B), indicating an endothelial capability. Moreover, immunofluorescent staining demonstrated that most of the cells were positive for both CD133 and VEGFR2 (Figure 1C), which further confirmed that those isolated cells were EPCs. Cells from passages 2‐4 were used for the following experiments.

### 3.2 IL‐1β up‐regulates CXCR7 expression in EPCs

**Figure 2:**
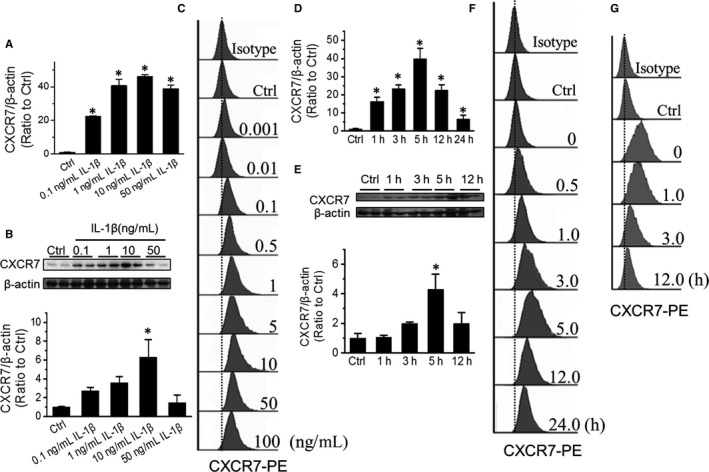
Dose‐ and time‐effect of IL‐1β‐induced CXCR7 expression. CXCR7 expression in EPCs treated with IL‐1β at different doses ranging from 0.001, 0.01, 0.1, 0.5, 1, 5, 10, 50 to 100 ng/mL for 5 h was analysed by qPCR(A), Western blot (B) and flow cytometry (C). CXCR7 expression in EPCs treated with 10 ng/mL IL‐1β for 0, 0.5, 3, 5, 12 or 24 h was analysed by qPCR (D), Western blot (E) and flow cytometry (F), and CXCR7 expression at 0, 1, 3 and 12 h after pre‐treatment with 10 ng/mL IL‐1β for 5 h (G) was detected by flow cytometry. Data shown in graphs represent three independent experiments. * *P* < .05, vs Control (Ctrl)

First, we investigated the dose effect of IL‐1β treatment on CXCR7 expression level. EPCs were treated with IL‐1β at 0.001, 0.01, 0.1, 0.5, 1, 5, 10, 50 or 100 ng/mL for 5 hours, and CXCR7 expression level was detected by real‐time PCR, Western blot and flow cytometry. The results showed that IL‐1β treatment enhanced CXCR7 expression in a dose‐dependent manner and that the most effective dose was 10 ng/mL (Figure 2A‐C). Second, we investigated the time‐course effect of IL‐1β treatment on CXCR7 expression level. EPCs were treated with 10 ng/mL IL‐1β for 0, 0.5, 3, 5, 12 or 24 hours, and CXCR7 expression level was detected by real‐time PCR, Western blot and flow cytometry. We found that IL‐1β treatment elevated CXCR7 expression level in a time‐dependent manner, reaching the peak at 3 hours and slightly regressing thereafter (Figure 2D‐F). In addition, we detected the time‐course regression of CXCR7 expression level after IL‐1β deprivation. EPCs were treated with IL‐1β for 5 hours; then, IL‐1β was removed, and CXCR7 expression was detected by flow cytometry at 0, 1, 3 and 12 hours later. The results showed that the CXCR7 expression level in EPCs was fairly maintained for 1 hour after deprivation of IL‐1β and then gradually regressed to baseline level within 12 hours (Figure 2G).

### 3.3 IL‐1β promotes capillary and tube formation in EPCs in a CXCR7‐dependent manner

**Figure 3:**
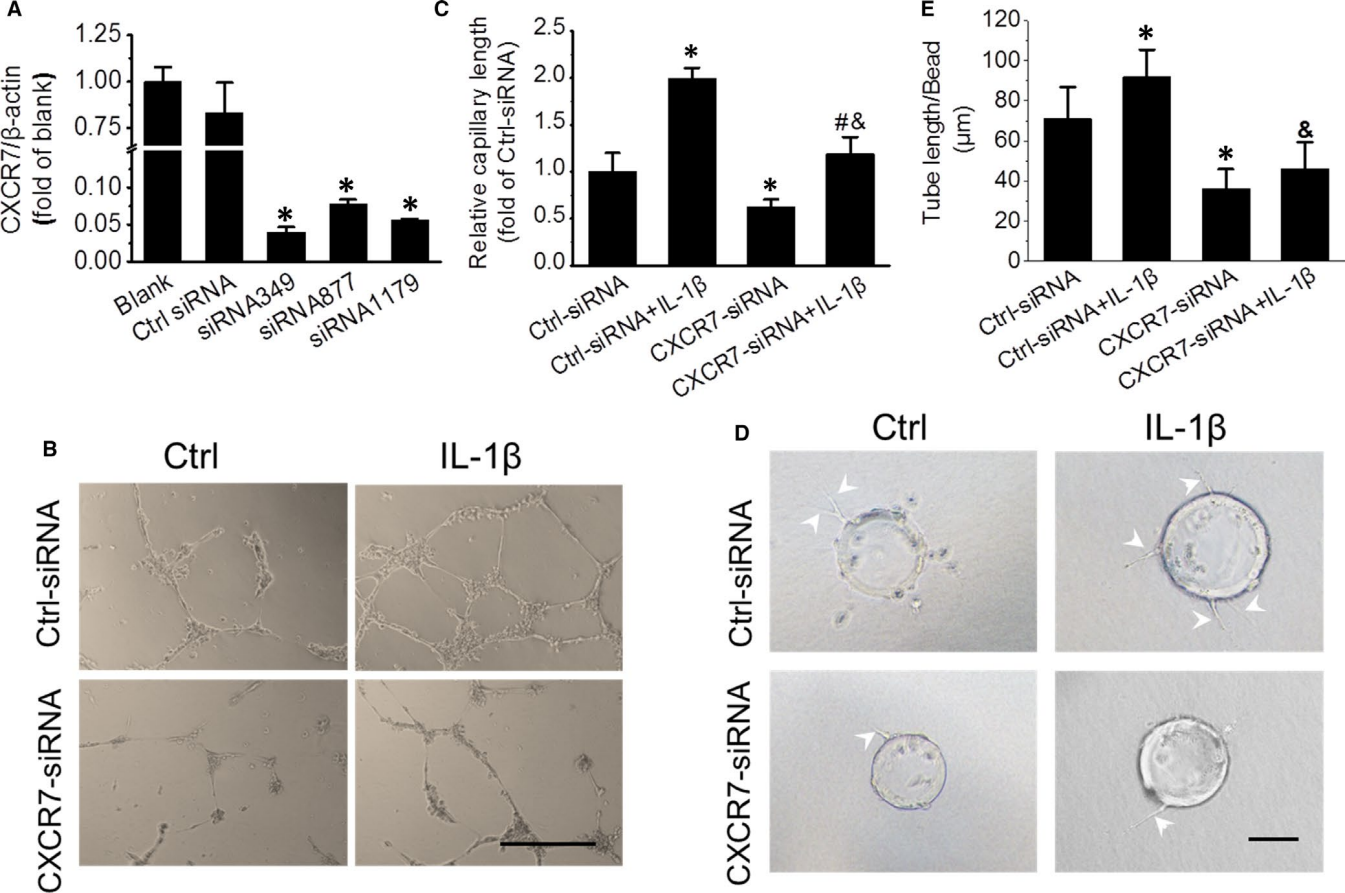
CXCR7 siRNA transfection impairs EPC tube formation induced by IL‐1β. CXCR7 mRNA expression in EPCs transfected with different siRNAs was detected by real‐time PCR (A). The effects of siRNA transfection on the capillary formation capability of EPCs with or without 10 ng/mL IL‐1β treatment were evaluated by Matrigel capillary formation assay (B), and the relative tube length was evaluated using ImageJ software (C). The effects of siRNA transfection on the tube formation capability of EPCs with or without 10 ng/mL IL‐1β treatment were evaluated by fibrin gel bead assay (D), and the relative tube length was also evaluated (E). Data shown in the graphs represent three independent experiments. * *P* < .05, vs Control (Ctrl); # *P* < .05, vs EPCs with control siRNA treatment; & *P* < .05, vs EPCs with CXCR7 siRNA treatment. Scale bar represents 100 μm

Considering the critical role of CXCR7 in EPC‐induced angiogenesis,^27^ we further investigated the role of CXCR7 in the IL‐1β‐mediated angiogenic capability of EPCs via Matrigel capillary formation assay and fibrin gel bead assay. For this purpose, CXCR7 was knocked down via CXCR7 siRNA transfection (Figure 3A). The Matrigel capillary formation assay showed that EPCs treated with IL‐1β formed longer capillaries than control EPCs, and siRNA interference with CXCR7 impaired the tube formation capability of EPCs under basal conditions and blocked IL‐1β‐stimulated tube formation (Figure 3B,C), indicating that CXCR7 plays a critical role in EPC tube formation in the presence and absence of IL‐1β stimulation. The fibrin gel bead assay showed a similar profile (Figure 3D,E). IL‐1β‐treated EPCs formed a greater number of tube‐like structures that were also longer and with branching, while CXCR7 knockdown substantially impaired tube‐like structure formation in the presence and absence of IL‐1β, which indicated that CXCR7 is involved in the enhanced tube formation capability of EPCs under IL‐1β treatment.

### 3.4 Inhibition of NF‐κB reduces IL‐1β‐mediated CXCR7 up‐regulation in EPCs

**Figure 4:**
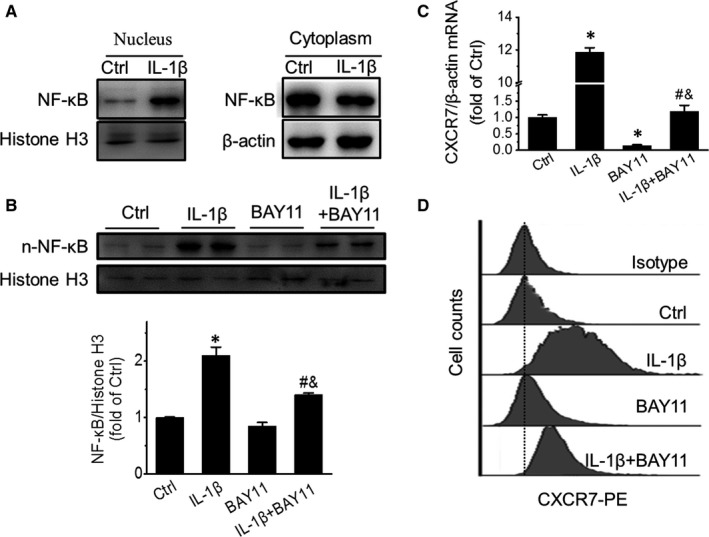
Inhibiting NF‐κB impairs CXCR7 up‐regulation induced by IL‐1β. Nuclear and cytoplasmic NF‐κB content in EPCs treated with or without 10 ng/mL IL‐1β for 5 h (A) in the presence or absence of 10 μmol/L BAY11‐7082 (B) was detected by Western blot. CXCR7 mRNA and protein expression of EPCs treated with IL‐1β in the presence or absence of BAY11‐7082 were detected by qRT‐PCR (C) and flow cytometry (D), respectively. Data shown in graphs represent three independent experiments. * *P* < .05, vs Control (Ctrl); # *P* < .05, vs EPCs with IL‐1β treatment; & *P* < .05, vs EPCs with BAY11‐7082 treatment

After confirming the role of CXCR7 in IL‐1β‐enhanced EPC tube formation, we further investigated the mechanism of how IL‐1β up‐regulates CXCR7 expression. NF‐κB is one of the typical downstream signalling molecules of IL‐1β.^34^ Therefore, we detected the direct effect of IL‐β on NF‐κB activation in EPCs. The results showed that IL‐1β stimulation significantly increased NF‐κB translocation from the cytoplasm to the nucleus, indicating that IL‐1β treatment induced NF‐κB activation in EPCs (Figure 4A). To determine the role of NF‐κB in IL‐1β‐enhanced CXCR7 expression in EPCs, BAY 11‐7082, a specific inhibitor of the nuclear translocation of NF‐κB, was used to investigate the direct link between IL‐1β stimulation, NF‐κB and CXCR7 up‐regulation. BAY11‐7082 (10 μmol/L) treatment had no significant effects on nuclear NF‐κB expression under basal conditions (0.85 ± 0.06 vs 1 ± 0.009, *P* > .05) but significantly inhibited IL‐1β‐stimulated NF‐κB nuclear translocation (1.40 ± 0.03 vs 2.10 ± 0.14, *P* < .05, Figure 4B). Under the same experimental conditions, BAY11‐7082 treatment almost completely abolished the IL‐1β‐mediated up‐regulation of CXCR7 at the mRNA level (1.19 ± 0.19 vs 11.86 ± 0.27, *P* < .05, Figure 4C), and significantly attenuated IL‐1β‐mediated CXCR7 up‐regulation on the cell surface of EPCs (Figure 4D). These results indicate that IL‐1β‐stimulated CXCR7 up‐regulation is at least partially mediated by IL‐1β‐stimulated NF‐κB nuclear translocation.

### 3.5 Erk1/2 is involved in IL‐1β‐induced tube formation of EPCs independent of the NF‐κB/CXCR7 pathway

**Figure 5:**
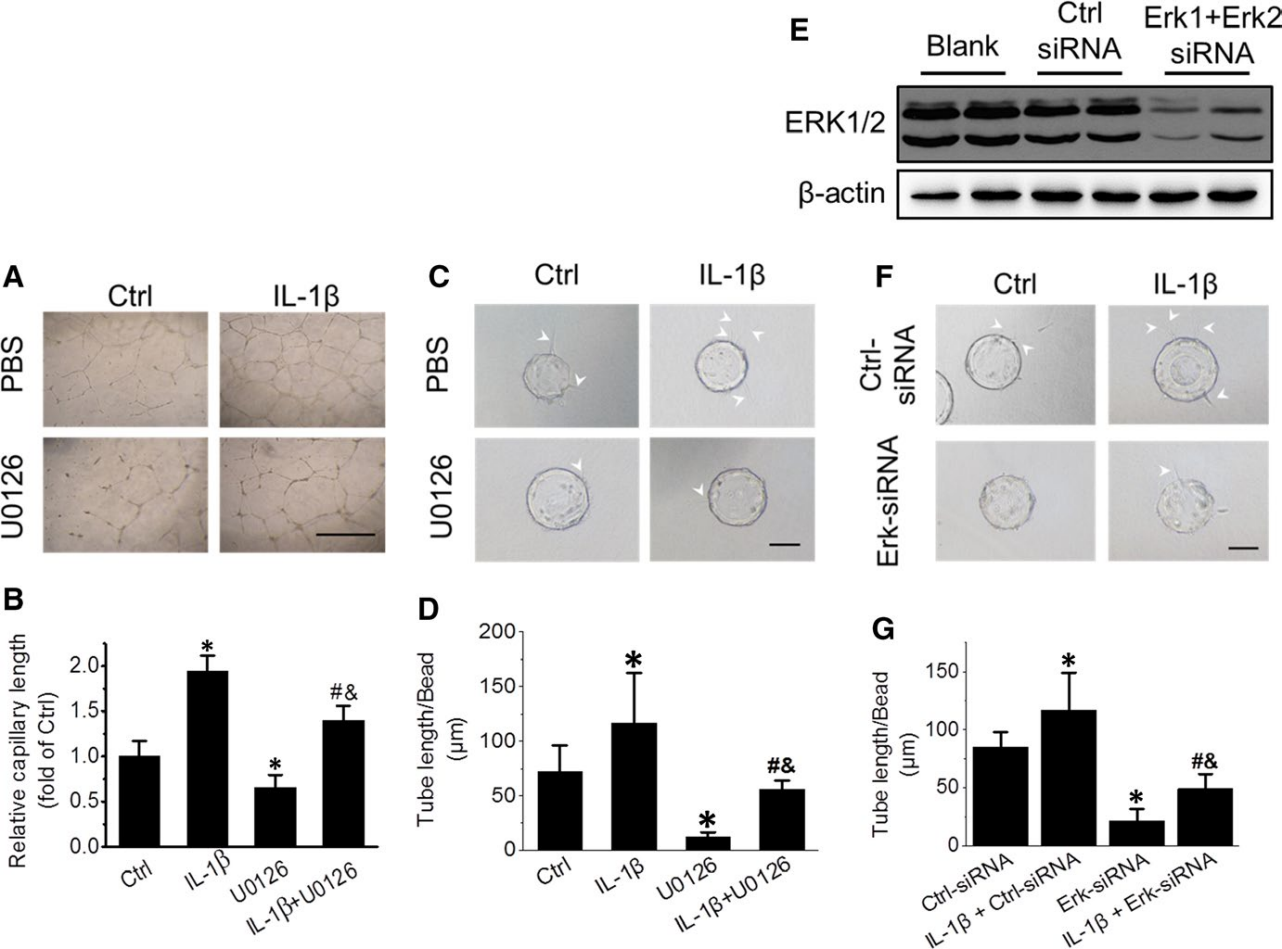
Inhibiting Erk1/2 activation impairs EPC tube formation. The effect of U0126 treatment on the IL‐1β‐induced angiogenic function of EPCs was evaluated by capillary formation assay (A and B) and fibrin gel bead assay (C and D). Erk1/2 expression in EPCs transfected with Erk1‐ and Erk2‐siRNA was detected by Western blot (E). The effect of blocking Erk1/2 expression by siRNA on the IL‐1β‐induced angiogenic function of EPCs was evaluated by fibrin gel bead assay (F and G). Data shown in graphs represent three independent experiments. * *P* < .05, vs Control (Ctrl); # *P* < .05, vs EPCs with IL‐1β treatment; & *P* < .05, vs EPCs with U0126 treatment. Scale bar represents 100 μm

Erk1/2 is another downstream signal mediator of IL‐1β and has been demonstrated to mediate IL‐1β‐stimulated angiogenesis in mouse EPCs in vitro.^32^ Herein, we further investigated whether Erk1/2 is involved in IL‐1β‐induced capillary formation and CXCR7 up‐regulation by using the Erk1/2 inhibitor U0126 and Erk siRNA transfection. Results showed that U0126 (10 μmol/L) treatment significantly impaired IL‐1β‐promoted EPCs capillary formation (Figure 5A,B). This result indicated that Erk1/2 is also involved in IL‐1β‐induced tube formation in EPCs, which was confirmed by the fibrin gel bead assay (Figure 5C,D). Moreover, siRNA transfection experiments also reached similar conclusions. Erk1/2 knockdown by Erk1‐ and Erk2‐siRNA transfection (Figure 5E) impaired the tube‐structure formation capability of EPCs in the presence or absence of IL‐1β (Figure 5F,G).

**Figure 6:**
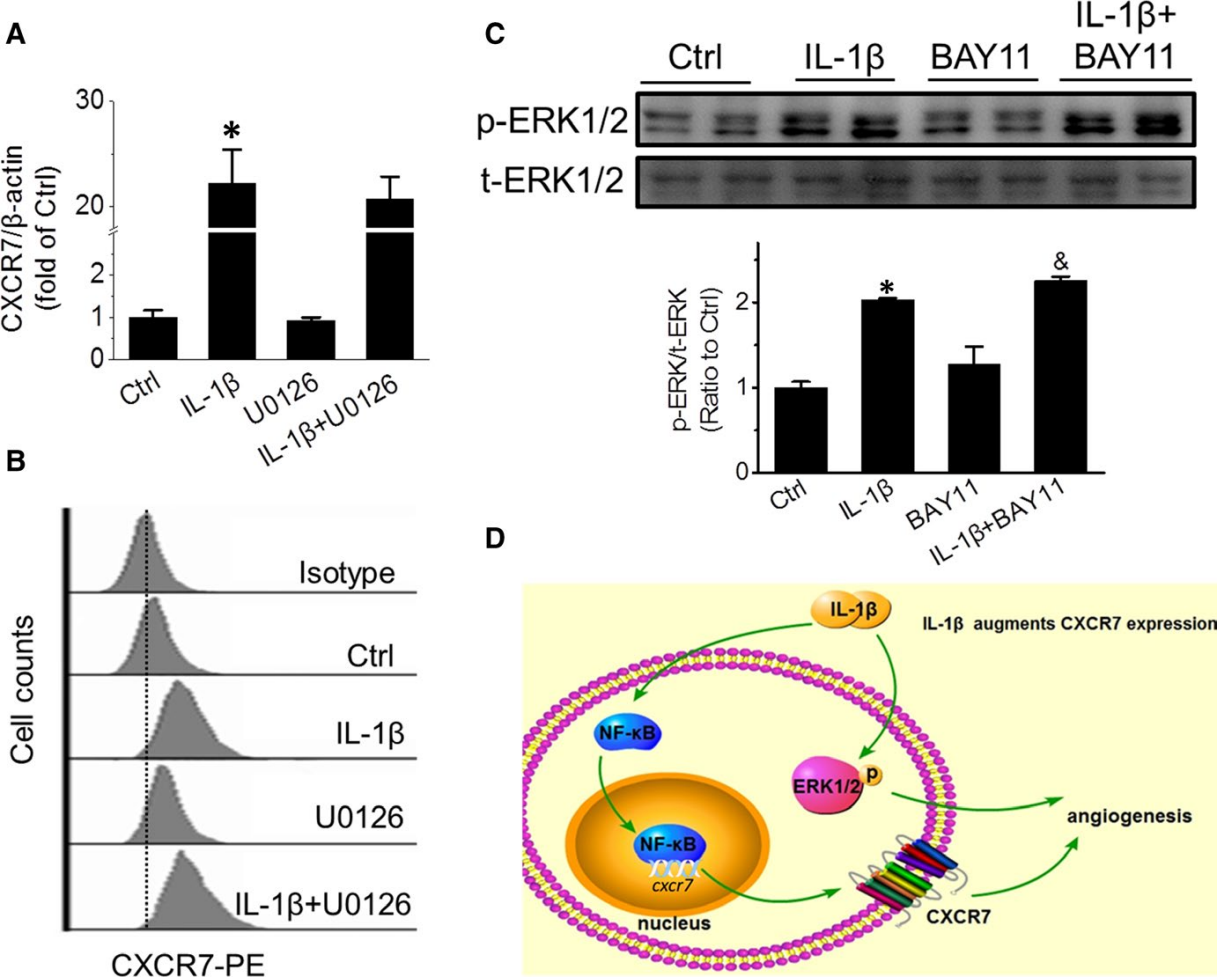
NF‐κB/CXCR7 and Erk1/2 independently mediate IL‐1β‐induced angiogenesis in EPCs. CXCR7 mRNA and protein expression in EPCs treated with 10 ng/mL IL‐1β in the presence or absence of 10 μmol/L U0126 were detected by qRT‐PCR (A) and flow cytometry (B), respectively. Erk1/2 phosphorylation in EPCs treated with IL‐1β in the presence or absence of BAY11‐7082 was detected by Western blot (C). IL‐1β promotes EPC angiogenesis via the NF‐κB/CXCR7 and Erk1/2 pathways, as represented in the schematic diagram (D). Data shown in graphs represent three independent experiments. * *P* < .05, vs Control (Ctrl); & *P* < .05, vs EPCs with BAY11‐7082 treatment

To determine whether Erk1/2 is involved in IL‐1β‐induced CXCR7 up‐regulation in EPCs, IL‐1β‐induced CXCR7 expression was detected with and without Erk1/2 inhibitor treatment. The results showed that U0126 treatment did not alter IL‐1β‐promoted CXCR7 expression at the mRNA level (20.77 ± 0.48 vs 22.20 ± 0.57, *P* > .05, Figure 6A) or at the protein level on the cell surface of EPCs (Figure 6B). To further determine whether Erk1/2 was regulated by the NF‐κB/CXCR7 signalling pathway, IL‐1β‐induced Erk1/2 phosphorylation was detected with and without the NF‐κB inhibitor BAY 11‐7082. The results showed that IL‐1β‐promoted phosphorylation of Erk1/2 was not affected by NF‐κB inhibition (2.26 ± 0.05 vs 2.04 ± 0.016, *P* > .05, Figure 6C). These results demonstrated that Erk1/2 is neither upstream nor downstream of the NF‐κB/CXCR7 signal pathway. NF‐κB/CXCR7 and Erk1/2 are two independent pathways involved in the IL‐1β‐promoted angiogenic capability of EPCs (Figure 6D).

## 4 DISCUSSION

It is well‐established that inflammation and angiogenesis are interdependent.^35^ Inflammatory factors have been found to play a principle role in pathological angiogenesis in numerous diseases, including rheumatoid arthritis, psoriatic diseases,^35^ diabetic retinopathy^36^ and tumorigenesis.^37^ As a typical inflammatory factor, IL‐1β has also been found to be involved in angiogenesis.^38^ IL‐1β can up‐regulate VEGF expression in tumour cells^39^ and angiopoietin‐1 expression in human endothelial cells.^31^ In this study, we demonstrated that IL‐1β promotes the angiogenic capability of EPCs via the NF‐κB/CXCR7 and Erk1/2 pathway.

Untreated EPCs only have a basal expression of CXCR7, but IL‐1β can induce CXCR7 expression in a dose‐ and time‐dependent manner (Figure 2). CXCR7 knockdown impaired IL‐1β‐promoted EPC tube formation (Figure 3). These results indicate that CXCR7 plays a critical role in IL‐1β‐induced EPC angiogenesis, which is consistent with the findings of Watanabe, K. and colleagues in HUVECs.^40^

NF‐κB is a classical IL‐1β‐induced downstream signalling molecule that plays critical roles in IL‐1β‐induced angiogenesis in mesenchymal stromal cells^41^ and angiogenic factor expression in temporomandibular disc cells.^34^ Moreover, NF‐κB and G protein‐coupled receptors are closely connected in inflammation and tumorigenesis.^42^ Herein, we found that the NF‐κB inhibitor BAY 11‐7082 can abolish IL‐1β‐induced CXCR7 expression in EPCs and even decreases the baseline CXCR7 mRNA expression level in EPCs (Figure 4C). These results indicate that NF‐κB plays a critical role in CXCR7 transcription induced by IL‐1β. However, how NF‐κB regulates CXCR7 expression needs to be further investigated.

Cancer is an angiogenesis‐dependent disease, and anti‐angiogenic drugs are considered to be potent in cancer therapy.^43^ IL‐1β can infiltrate and promote angiogenesis in solid tumours,^30^ and the mitogen‐activated protein kinase (MAPK) pathway also plays a principle role in tumour angiogenesis.^44^ Rosell et al^32^ found that IL‐1β augmented the angiogenic response of murine EPCs in an Erk1/2‐dependent manner, which is consistent with our results in this study (Figure 5). Moreover, we also demonstrated that NF‐κB/CXCR7 and Erk1/2 are two independent pathways mediating EPC angiogenesis induced by IL‐1β, which is consistent with the research of Hartmann.^45^The MAPK pathway is an important target for inhibiting tumour progression and angiogenesis,^32^ and our results in the present study suggest that antagonizing CXCR7 might be a potent complementary strategy for inhibiting MAPK during anti‐angiogenic therapy in cancer.

In some other pathological conditions, such as diabetes, angiogenesis is impaired and improving EPC function has been considered as an effective approach to ameliorate angiogenesis.^46^ Our recent study^4^ showed that enhancing CXCR7 expression in EPCs via lentiviral transfection can promote EPC angiogenic capacity and ameliorate blood perfusion in diabetic ischaemic hindlimbs. Considering the potential biosafety risk in lentiviral transfection, finding a safer way to up‐regulate CXCR7 expression in EPCs would be beneficial for ischaemic vascular diseases. In the present study, IL‐1β was found to substantially up‐regulate CXCR7 expression in EPCs in a NF‐κB‐dependent manner (Figures 2 and 4). In addition to IL‐1β, another pro‐inflammatory cytokine, lipopolysaccharide (LPS), was also found to be able to increase proliferation, migration and tube formation of choroidal endothelial cells through TLR4/NF‐κB‐mediated up‐regulation of CXCR4 and CXCR7.^47^ These results reveal a potential way to enhance CXCR7 expression in EPCs and may provide some enlightenment for the further development of CXCR7 agonists.

## CONFLICT OF INTEREST

The authors confirm that there are no conflicts of interest.

## AUTHOR CONTRIBUTION

Xia Fan, Luqing He and Xue Meng performed flow cytometry, Matrigel capillary formation assay and fibrin gel bead assay; Qiaoxia Dai, Junhong He and Xiaozhen Dai performed EPCs isolation and identification. Xiangjuan Chen and Lin Lin obtained the cord blood samples. Chi Zhang, Da Sun, Shiyue Sun, Jun Chen and Liangmiao Chen performed siRNA transfection, qPCR and Western blot. Xiaoqing Yan designed the experiments, processed data and wrote the manuscript. Yi Tan revised the paper.

## Data Availability

The datasets generated during and/or analysed during the current study are available from the corresponding author upon reasonable request.
